# Burning Mouth Syndrome (BMS)—Treatment with Verbal and Written Information, B Vitamins, Probiotics, and Low-Level Laser Therapy: A Randomized Clinical Trial

**DOI:** 10.3390/dj10030044

**Published:** 2022-03-10

**Authors:** Božana Lončar-Brzak, Ivana Škrinjar, Vlaho Brailo, Danica Vidović-Juras, Lada Šumilin, Ana Andabak-Rogulj

**Affiliations:** 1 Department of Oral Medicine, School of Dental Medicine, University of Zagreb, 10000 Zagreb, Croatia; loncar@sfzg.hr (B.L.-B.); iskrinjar@sfzg.hr (I.Š.); brailo@sfzg.hr (V.B.); djuras@sfzg.hr (D.V.-J.); 2 Department of Oral Medicine, University Clinical Hospital Zagreb, 10000 Zagreb, Croatia; 3 Polyclinic Aviva, 10000 Zagreb, Croatia; lada.sumilin@poliklinika-aviva.hr

**Keywords:** burning mouth syndrome, low-level laser therapy, B vitamins, probiotics, oral health impact profile, visual analogue scale

## Abstract

Background: The objective of this study was to determine the most effective treatment option for burning mouth syndrome. Methods: Informative treatment alone, B vitamin injections, oral cavity probiotics, and low-level laser therapy were evaluated and compared. The study included new patients diagnosed with burning mouth syndrome, who were randomly allocated into one of four treatment groups. The primary outcome was improvement in patient’s quality of life as determined by a self-perceived Oral Health Impact Profile-14 (OHIP-14) quality of life questionnaire before and after therapy. The secondary outcome was determination of mucosal symptom intensity according to visual analog scale (VAS) grading from 0 to 10. Data were submitted to statistical analysis. Results: A total of 62 patients completed the study. Oral cavity probiotics and LLLT scores for OHIP-14 resulted in a statistically significant difference before and after therapy. Standardized effect sizes between OHIP scores before and after treatment were the greatest for patients who had received oral cavity probiotics. Conclusions: Oral cavity probiotics and LLLT were the most effective treatment for improvement in quality of life. Further investigation on a larger group of patients is required.

## 1. Introduction

Burning mouth syndrome (BMS) is characterized by idiopathic burning or painful sensations of oral mucosa with clinically unchanged appearance. Despite numerous proposed definitions in the literature, there is still no universally accepted definition for BMS and no proper diagnostic criteria [1]. Two clinical forms of BMS were proposed by Scala et al., namely primary BMS, which is defined as burning sensation of the oral mucosa without any local or systemic causes, and secondary BMS, which may be a result of some local or systemic causes [2]. These causes include oral infections, hyposialia, allergies, nutritional deficiencies, endocrine disorders, administration of some drugs, oral mucosal diseases such as oral lichen planus, etc. [3]. The diagnosis is established by exclusion of local and systemic potential causes [2,4]. In the general population, the reported prevalence of this unpleasant condition is less than 1–3.7% [5]. However, it affects almost one-third of postmenopausal women [6]. The numbers in the literature vary greatly due to nonunique criteria for diagnosis [2]. Clinicians are often not sufficiently aware of this condition, so it is difficult to estimate its real prevalence in the general population. Patients usually seek help from different medicine and dental medicine specialists and undergo a variety of laboratory tests and examinations, usually without improvement in the symptoms. A factor patients often have in common is that they are currently under stress or have recently had a stressful life situation. The condition itself increases the patients’ level of concern about their health. Results from the literature have shown that these patients are prone to depression and anxiety [7,8], so the first step in treatment should be explanation and reassurance about the unpleasant but benign nature of the condition. The diagnosis is established based on characteristic description of the symptoms, oral mucosa examination, and laboratory tests to exclude potential nutritional deficits.

A universally accepted treatment protocol for BMS does not exist. The literature offers results of different therapeutic approaches, but they have limited effectiveness and it is difficult to make comparisons between the approaches [9,10,11,12,13,14]. Our first line of treatment for these patients is a detailed verbal and written explanation about the nature of the disease. For some patients, this treatment is sufficient, but others search for additional therapeutic options. Several studies have demonstrated positive effect of low level laser therapy (LLLT) in the treatment of BMS [14,15] due to the analgesic, anti-inflammatory, and repairing effect of LLLT on tissues [14,15,16]. LLLT is a noninvasive treatment that is well accepted by patients. However, the results in the literature are not unanimous and vary greatly according to the applied parameters, the pain scales used, and reporting practices [15]. Further research is needed to assess the effect of LLLT in the treatment of BMS.

Some authors have shown that patients with BMS may have decreased levels of B1, B6, and B12 vitamins [17]. It has been shown that B1, B6, and B12 especially have different neurospecific functions and are important for maintenance of normal neurological functions in the peripheral and central nervous systems [18]. B vitamins are a frequent therapeutic choice for neuropathies [19] and might be used as BMS treatment [20] as evidence points to a neuropathic background in BMS [2].

Recently, some probiotics have been proposed as being of help in BMS due to an increase in “good” oral and gut bacteria (not published data), although no studies with their application for BMS have been published. Data from the literature show that *Lactobacillus reuteri* from BioGaia Prodentis has antimicrobial effect on periodontal pathogens [21,22,23]. The metabolic activity of probiotic cultures releases different bioactive metabolites, which are called postbiotics. They can have a direct or indirect beneficial effect on health due to their immunomodulatory, anti-inflammatory, antioxidant, and anticancer properties [24]. Results of an in vitro study have shown that *Lactobacilli* postbiotics inhibit biofilm formation and potentially disrupt *Aggregatibacter actinomycetemcomitans* colonization [25]. The only in vivo study with results published so far is a randomized clinical trial that showed that oral gel with postbiotic content is as effective as a conventional chlorhexidine gel for the periodontal parameters observed, in addition to scaling and root planning [26]. Therefore, postbiotics present a novel therapeutic option for periodontal disease, although they have not yet been tested for BMS. Moreover, oral probiotics might stimulate the gustatory system and decrease pain [27]. One of the possible explanations regarding BMS etiology is that the chorda tympani and the lingual nerve mutually inhibit impulse conduction to the brain, so the stimulation of taste blocks painful input from the lingual nerve [28]. A characteristic finding in BMS patients is that food and drink intake decreases burning sensations and painful symptoms [29].

Researchers have achieved positive results with some types of therapy, such as LLLT and B vitamin injections [11,12]. Therefore, we decided to compare the aforementioned treatments with our first line of treatment. As BMS is a condition that impairs quality of life, the oral health impact profile questionnaire (OHIP-14) validated in Croatia [30] was used to assess quality of life relating to oral health. The primary aim of this study was to determine the effect of treatment on quality of life, while the secondary aim was to determine the effect of treatment on intensity of subjective symptoms. The effectiveness of the following four therapeutic options for BMS patients were evaluated: verbal and written information about the condition alone, B vitamin injections, oral cavity probiotics, and LLLT. Null hypothesis was that verbal and written information, B vitamin injections, oral probiotics, and LLLT are equally effective in the treatment of BMS.

## 2. Materials and Methods

This study was approved by the Ethical Committee of the School of Dentistry, University of Zagreb, Croatia (approval number: 05-PA-30-9/2018). The research is registered at the U.S. National Institutes of Health, ClinicalTrials.gov (trial identifier: NCT04475614). Each patient signed an informed consent according to the Declaration of Helsinki. Each patient was clearly explained about the diagnosis and given an information leaflet. At the beginning of the study, they filled out a self-perceived quality of life questionnaire (Oral Health Impact Profile; OHIP-14) and determined the intensity of mucosal symptoms according to the visual analog scale (VAS) grading from 0 to 10 (0 = without symptoms, 10 = the worst possible symptoms), which was also repeated at the check-up one month after the treatment ended. After inclusion in the study, patients were randomly allocated into one of four groups by a random number generator and then assigned to interventions. After allocation into one of four groups, neither the participants nor the care providers were blinded.

### 2.1. Eligibility Criteria

The participants were patients at the Department of Oral Medicine, School of Dental Medicine, University of Zagreb, Croatia, and were enrolled in the study by specialists of oral medicine. The inclusion criteria was newly diagnosed burning sensations of oral mucosa that lasted at least three months before treatment, without clinical changes in oral mucosa. All patients had proper laboratory findings of complete blood count, iron, and blood glucose. None of them were taking angiotensin-converting enzyme inhibitors. Exclusion criteria were patients who had local or systemic causes of BMS; had received any type of treatment for BMS before this one; were under 18 years of age; had impaired laboratory findings of complete blood count, iron, and blood glucose; or had anamnestic data about taking angiotensin-converting enzyme inhibitor.

### 2.2. Study Setting

This was an open-label randomized controlled trial carried out at an outpatient clinic of a major Croatian tertiary academic center.

### 2.3. Interventions

The first group of patients were given verbal explanation and an information leaflet only; the second group of patients were given verbal explanation, an information leaflet, and vitamin B injections (Neurobion, Merck, Darmstadt, Germany); the third group of patients were given verbal explanation, an information leaflet, and oral cavity probiotics (BioGaia Prodentis, BioGaia AB, Stockholm, Sweden); and the fourth group of patients were given verbal explanation and an information leaflet and were then treated with LLLT. Treatments and components of the products used in this study are shown in Table 1.

**Figure 1:**
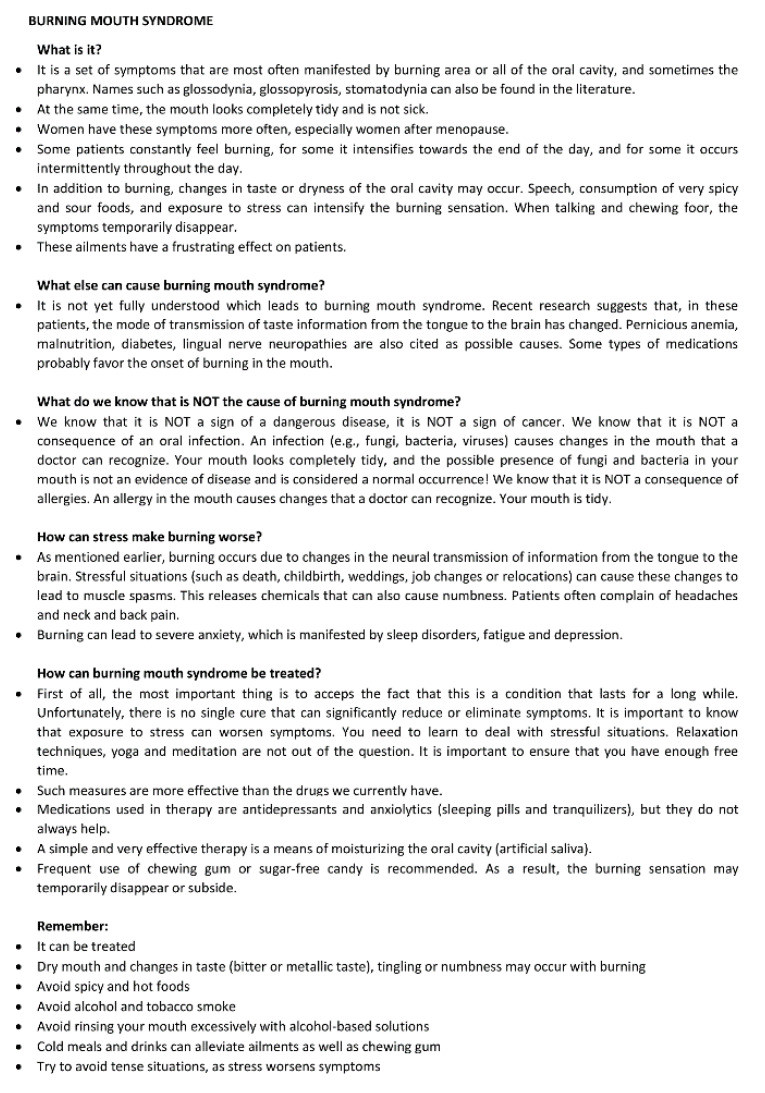
Written information for patients with BMS translated to English.

Verbal explanation was carried out by a thorough and informative conversation about the condition. After the conversation, an information leaflet was given to the patient to read at home. The information leaflet was translated and adapted according to the leaflet written by the Facial Pain Team based at the Royal National ENT and Eastman Dental Hospitals. Translation of our information leaflet is shown in Figure 1.

Solution for the B vitamin injection contained 100 mg of vitamin B1 and B6 and 1 mg of vitamin B12 (Neurobion, Merck, Darmstadt, Germany) in 3 mL of aqueous solution. Each patient received nine vitamin B injections every other day into the gluteal muscle (i.m.) for three weeks, excluding weekends.

Patients who received oral probiotics (BioGaia Prodentis, BioGaia AB, Stockholm, Sweden) were instructed to melt one lozenge in the mouth every evening after flossing and toothbrushing for one month. One lozenge contains the patented lactic acid bacterium *Lactobacillus reuteri* Prodentis^®^ (*L. reuteri* DSM 17938 and *L. reuteri* ATCC PTA 5289).

LLLT was carried out with a wavelength of 685 nm from Ga–Al–As diode type of laser (BTL2000 Medical Technologies, s.r.o., Prague, Czech Republic) on three reported burning sites. The LLLT patients received a total of 10 treatments once a day for 10 consecutive days, excluding weekends. The laser parameters are shown in Table 2.

### 2.4. Primary Outcome Measure

The primary outcome measure was improvement in patient’s quality of life as determined by a self-perceived quality of life questionnaire (Oral Health Impact Profile; OHIP-14) [30], which was filled out after inclusion in the study (before treatment) and at the control examination one month after the end of treatment. Quality of life was established based on the sum of participants’ answers to the 14 questions. The answer options with their respective values were as follows: 0 = never, 1 = rarely, 2 = sometimes, 3 = repeatedly, 4 = always, with a maximum score of 56. The higher the score, the worse the quality of life.

### 2.5. Secondary Outcome Measures

The secondary outcome measure was improvement in patient’s subjective burning symptoms as measured on a visual analogue scale (VAS) grading from 0 to 10 (0 = without symptoms, 10 = worst possible symptoms) [31]. Burning symptoms were determined on the VAS scale after inclusion in the study (before treatment) and at the control examination one month after the end of treatment.

### 2.6. Sample Size Determination

Sample size was calculated from the literature data [14] by power analysis with significance level α = 0.05 and power β = 0.8. With expected mean difference of 0.30, the minimal number of patients in each group was estimated to be 11.

### 2.7. Allocation

After inclusion in the study, patients were randomly allocated into one of four groups by a random number generator and then assigned to interventions.

### 2.8. Blinding

After allocation into one of four groups, neither the participants nor the care providers were blinded.

### 2.9. Statistical Analysis

Statistical analysis was performed by MedCalc statistical software, version 18.10.2. (Ostend, Belgium). Distribution of data was tested by the Kolmogorov–Smirnov test, which showed that distribution was not normal. Therefore, nonparametric statistics were used. Differences between groups regarding age and sex were tested by Kruskal–Wallis test. The Mann–Whitney *U* test was used to determine comparison of OHIP and VAS percentage decrease between groups. Wilcoxon signed-rank test for related samples was used to compare differences between OHIP scores and VAS scores before and after treatment. Level of significance in all tests was 0.05 (*p* < 0.05). Standardized effect size was calculated for OHIP-1 and OHIP-2 in patients with different types of therapy.

## 3. Results

**Figure 2:**
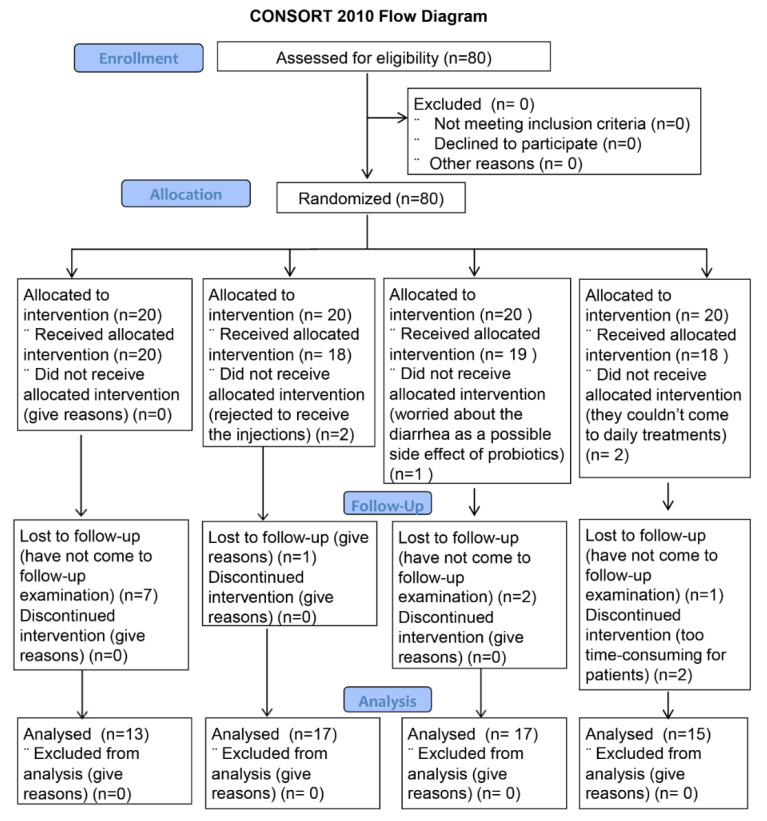
Flow chart of the enrollment of participants.

A total of 80 patients were included and randomized into four treatment groups, and 62 patients finished their treatment and came to the follow-up control examination (Figure 2). They were recruited in the period of six months (from September 2018 to March 2019) and followed up one month after the end of treatment. A total of 13 patients were given verbal explanation and information leaflet only, 17 patients were given vitamin B injections, 17 patients received oral cavity probiotics, and 15 patients were treated with LLLT. There was no statistically significant difference in age and gender between the groups (*p* = 0.99) (Table 3). None of the participants reported side effects during or after any of the treatment. The percentage decrease in the OHIP and VAS values between groups was measured by intergroup comparison using the Mann–Whitney test. No significant differences between the groups were found (Table 4 and Table 5). Statistically significant differences in OHIP scores before and after therapy were detected for oral cavity probiotics and LLLT. Differences in OHIP scores before and after different types of therapy showed significance for oral cavity probiotics and LLLT. Standardized effect sizes between OHIP scores in patients with different types of therapy showed that oral cavity probiotics were the most effective treatment for improvement in quality of life (Table 6). Differences in VAS scores before and after different types of therapy showed significance for all types of treatment (Table 7).

## 4. Discussion

The literature offers a variety of treatment options for BMS [9,10,11,12,13,14,15], but none of them are universally accepted. Many studies have shown that BMS is a neuropathic condition involving the peripheral and/or central nervous systems [2,32], but choosing adequate treatment is challenging. Although the condition is not threatening, it increases the level of stress in patients as well as medical costs for examinations and unsuccessful therapies.

Education of patients is important for their understanding of the diagnosis, but some of them, even after elucidating the benign nature of their symptoms, seek some therapeutic option and feel better if they receive one. The fact that there is no universal cure does not mean that different treatment options should not be tried and evaluated. Individualized approach to these patients has been suggested [33] in order to achieve more patient-specific treatment. This is confirmed by the results of this study, which showed that all the tested treatment options improved patient symptoms, although only some treatment options also improved their quality of life. To enable easier comparison of the results of different studies, Liu et al. [10] suggested the use of a standardized 1 to 10 point VAS scale for patients’ symptoms, a validated quality of life survey, and a leaflet for patients containing information about their condition. These are all parameters we used in our study. In their review, the authors excluded studies without a placebo group [10]. A placebo group was not included in this study, but one of the groups received only verbal and written information about the condition without other treatment. Conclusions from the literature [10,33] point out the importance of patient education and correct information about their condition while also indicating the lack of a treatment option that would be efficient in all patients. Patients who do not receive detailed explanation about their condition continue to search for a cause for their problems.

In general, LLLT is used in medicine due to its biomodulating action and ability to penetrate the tissue [34]. Furthermore, literature data have shown the analgesic, anti-inflammatory, and repairing effect of LLLT on tissues [15,35]. LLLT inhibits nociceptive mediators, such as bradykinin and histamine, and releases analgesic substance, such as endorphins, which is responsible for its analgesic effect [16]. LLLT also blocks the depolarization of C-fibers, which transmit heat and pain stimuli, and amplifies ATP synthesis [15], which contributes to pain reduction. Recently published results have shown that LLLT affects microcirculation, reducing the capillary diameter and inflammatory vasodilatation and improving symptoms in BMS patients [36]. Up to now, LLLT has shown to be effective in the treatment of BMS [15,37]. The results of this study showed that some treatment options, such as oral cavity probiotics and LLLT, besides relief of symptoms, also improved quality of life. Oral cavity probiotics were even slightly more effective than LLLT in improving quality of life. Previous results of LLLT for treatment of BMS have shown that the treatment alone is effective for decreasing the level of stress and salivary cortisol in these patients [11], which is in accordance with the results of the impact of LLLT on quality of life of patients with BMS. The positive effect of LLLT on the intensity of symptoms and quality of life has also been reported in other studies [14,38], while some authors observed only improvement of symptoms without improvement in quality of life [39]. Studies published so far that have evaluated the use of LLLT in treatment of BMS have used different laser parameters. Great variations in the wavelength (630–980 nm), energy dose per treatment point (0.4–6 J), power output (20–300 mW), energy density of the laser (0.53–176 J/cm^2^), exposure time (10 s to 15 min), and number of laser treatments ranging from 1 to 20 can be seen [15]. Although they have reported positive effects of LLLT, it is difficult to compare the results, and there is still no consensus regarding the ideal laser parameters for this diagnosis.

In the literature, no published results of oral probiotics treatment of BMS was found, although some probiotics claim to help with BMS due to an increase of “good” oral and gut bacteria (not published data). Data from the literature show that *Lactobacillus reuteri* from BioGaia Prodentis has antimicrobial effect on periodontal pathogens [21,22,23] and that postbiotics present a novel therapeutic option for periodontitis [25,26]. It is known that postbiotics have a direct or indirect beneficial effect on health due to their immunomodulatory, anti-inflammatory, antioxidant, and anticancer properties [24], but their effect in BMS has not yet been tested. In these patients, it is possible that oral probiotics are efficient due to gustatory and mechanical stimulation of oral mucosa. Evidence from the literature indicates the link between BMS and gustatory system [27,28,29,40,41,42,43,44]. It is known that the burning symptoms are most pronounced in the evening and that oral intake of food and drinks, especially mint-flavored ones, decreases the symptoms [29,39].

Data from the literature have shown that high-dose B vitamin supplementation reduces depressive state, stress, anxiety, and tiredness and is important in prevention and maintenance of brain health and cognitive function [18,19,45,46,47]. It is known that depression and anxiety are a frequent finding among BMS patients [7,8]. B vitamins are also useful in neuropathic pain. Vitamin B12 reduces damage of the nervous fiber, while B1 and B6 have antinociceptive and antihyperalgesic effects [48]. Yet, results from the literature regarding the effect of B vitamins in the treatment of BMS are conflicting [20,49]. Our previous experience with B vitamin injections for BMS was very positive, but our objective then was only improvement of symptoms on a VAS scale [12], which is also confirmed with these results. Until now, we have not compared the effect of these therapeutic option on patients’ symptoms and quality of life. It is interesting that B vitamin injections was shown to be less potent in improving quality of life than oral probiotics, LLLT, and even informative treatment alone, although they were successful in relieving symptoms. It seems that patients are more prone to noninvasive treatment, which could explain our results.

The limitation of our results is the lack of a placebo group and a relatively short follow-up period of one month after the end of treatment regardless of the type of treatment. Future studies should try to include a placebo group and have a longer follow-up period. The advantage of our study is the absence of permanent or strong side effects reported by patients after any of the applied treatment options (only patients that received B vitamin injections reported mild and transient discomfort at the place of injection). Another advantage of the study is simultaneous comparison of four treatment options and their effect on patients’ symptoms and quality of life. Based on our results, we may assume that patients respond better to noninvasive treatment, such as oral probiotics and LLLT. Moreover, we have shown that even informative treatment alone improves quality of life by reassuring patients that there is no concern for their oral health. We recommend the use of information leaflet for patients with this diagnosis.

## 5. Conclusions

Oral cavity probiotics and LLLT were the most effective for improving quality of life in patients with BMS when compared to other treatment groups. Further investigation on a larger group of patients, including a placebo group, is required.

## Figures and Tables

**Table 1:** Treatments and components of the products used in this study.

Type of the Treatment	Informative Conversation and Translated and Adapted Leaflet (Facial Pain Team Based at the Royal National ENT and Eastman Dental Hospitals)	Neurobion Injections (100 mg of Vitamin B1 and B6 and 1 mg of Vitamin B12)	Oral Probiotics (BioGaiaProdentis, BioGaia AB, Stockholm, Sweden) One Lozenge Contains the Patented Lactic Acid Bacterium *Lactobacillus reuteri* Pro-Dentis^®^ (*L. reuteri* DSM 17938 and *L. reuteri* ATCC PTA 5289)	LLLT (Aluminium Gallium Arsenide Laser; Ga–Al–As Diode Type of Laser)
Application mode	At the first appointment.	Total of nine vitamin B injections every other day into the gluteal muscle (i.m.), for three weeks, excluding weekends.	One lozenge to melt in the mouth every evening after flossing and toothbrushing for one month.	Applied on three reported burning sites, with a total of 10 treatments once a day for 10 consecutive days, excluding weekends.

**Table 2:** Relevant technical data for low-level laser therapy (BTL2000 Medical Technologies, s.r.o., Czech Republic).

Parameter	Value
Wavelength (nm)	685
Dose (J/cm^2^)	2.0
Power (mW)	30
Power density (W/cm^2^)	0.003
Single treatment duration (s)	381
Distance (cm)	0.5
Treated surface area (cm^2^)	3
Frequency (Hz)	50
Cumulative dose (J/cm^2^)	60
Number of treatments	10

**Table 3:** Age and gender distribution among the different therapeutic groups (*p* > 0.05, Kruskal–Wallis test).

Treatment	Age (Median, Range) (Years)	Sex (F = Female, M = Male)
Informative	55 (38–83)	10 F, 3 M
B vitamins	62 (39–60)	13 F, 4 M
Oral probiotics	67 (43–84)	13 F, 4 M
LLLT	60 (44–83)	11 F, 4 M
*p*	>0.05	>0.05

**Table 4:** Intergroup comparison of the percentage decrease in OHIP values (*p* > 0.05, Mann–Whitney *U* test).

Intergroup Comparison (OHIP)	z-Score	*p*-Value
Informative: B vitamins	−0.23	0.409
Informative: probiotics	0.502	0.617
Informative: LLLT	−0.102	0.92
B vitamins: probiotics	−1.016	0.307
B vitamins: LLLT	−0.669	0.502
Probiotics: LLLT	0.314	0.756

**Table 5:** Intergroup comparison of the percentage decrease in VAS values (*p* > 0.05, Mann–Whitney *U* test).

Intergroup Comparison (VAS)	z-Score	*p*-Value
Informative: B vitamins	0.146	0.88
Informative: probiotics	0.921	0.357
Informative: LLLT	0	1
B vitamins: probiotics	−0.499	0.617
B vitamins: LLLT	0.460	0.645
Probiotics: LLLT	1.001	0.317

**Table 6:** Differences in OHIP scores before (OHIP-1) and after (OHIP-2) different types of therapy (Wilcoxon signed-rank test).

	Informative	B Vitamins	Oral Probiotics	LLLT
OHIP-1, median (range)	13 (3–24)	24 (0–48)	22 (2–50)	20 (4–35)
OHIP-2, median (range)	11 (0–24)	16 (0–51)	12 (0–46)	14 (2–31)
N	13	17	17	15
*p*	0.12	0.42	0.003	0.006
Standardized effect size	0.45	0.17	0.62	0.58

**Table 7:** Differences between VAS-1 and VAS-2 scores in BMS patients with different types of therapy (Wilcoxon signed-rank test).

	Informative	B Vitamins	Oral Probiotics	LLLT
VAS-1, median (range)	5 (3–7)	7 (3–10)	6 (2–10)	6 (4–9)
VAS-2, median (range)	3 (0–6.5)	4 (0–10)	5 (0–7)	5 (3–7)
N	13	17	17	15
*P*	0.001	0.003	0.004	0.004
